# Transvaginal Ultrasound vs. Magnetic Resonance Imaging: What Is the Optimal Imaging Modality for the Diagnosis of Endometriosis?

**DOI:** 10.3390/biomedicines11102609

**Published:** 2023-09-23

**Authors:** Alexandra Irma Gabriela Baușic, Daniela Roxana Matasariu, Andrei Manu, Elvira Brătilă

**Affiliations:** 1 Department of Obstetrics and Gynecology, Doctoral School, “Carol Davila” University of Medicine and Pharmacy, 020021 Bucharest, Romania; andrei.manu@rez.umfcd.ro; 2 Department of Obstetrics and Gynecology, “Carol Davila” University of Medicine and Pharmacy, 020021 Bucharest, Romania; elvira.bratila@umfcd.ro; 3 Department of Obstetrics and Gynecology, “Prof. Dr. Panait Sîrbu” Obstetrics and Gynecology Hospital, 060251 Bucharest, Romania; 4 Department of Obstetrics and Gynecology, University of Medicine and Pharmacy “Gr. T. Popa”, 700115 Iasi, Romania

**Keywords:** deep infiltrating endometriosis, non-invasive diagnosis, non-invasive imaging modality, optimization of surgical approaches, magnetic resonance imaging, transvaginal ultrasound, laparoscopy

## Abstract

Endometriosis, an intriguing gynecological illness, poses a substantial health concern for women of reproductive age, despite its widespread occurrence and limited comprehension. The objective of this study is to assess the diagnostic precision of transvaginal sonography (TVS) and pelvic magnetic resonance imaging (MRI) for the identification of deep infiltrated endometriosis (DIE). This study encompassed a cohort of 256 patients exhibiting signs and symptoms of endometriosis, with the aim of assessing the diagnostic accuracy over a span of four years. Both TVS and pelvic MRI were employed in the same centers to analyze each subject. The histopathologic analysis and laparoscopy were the most reliable and widely accepted methods for evaluation. TVS is a reliable diagnostic tool for ovarian endometriomas, obviating the necessity for MRI confirmation. The specificity of TVS in diagnosing ovarian endometriomas is 57.14%, while its sensitivity is 93.78%, resulting in an overall accuracy of 84.47%. In relation to parametrial lesions, the sensitivity, specificity, and accuracy of TVS and MRI were as follows: TVS: 9%, 97%, 32%, MRI: 27.14%, 89.19%, and 40.11%. Concerning the uterosacral lesions, the sensitivity, specificity, and accuracy of TVS and MRI were as follows: TVS:14.63%, 94.74%, and 55%, while MRI: 65.88%, 66.30%, and 66.1%. Regarding rectal endometriosis, the sensitivity, specificity, and accuracy of TVS and MRI were as follows: 69.72%, 76.87%, and 73.82% for TVS, and 66.28%, 94.51%, and 80.79% for MRI. The results of the present study indicate that whereas MRI generally exhibits superior capability in assessing the severity of endometriosis, TVS demonstrates sufficient diagnostic accuracy in DIE comparable to MRI.

## 1. Introduction

Endometriosis, a benign gynecological condition, is a notable health concern among women of reproductive age, despite its high prevalence and limited understanding. Endometriosis is a medical ailment that is influenced by estrogen levels and predominantly impacts women of reproductive age. It is infrequently observed prior to puberty or following menopause. Endometriosis affects around 6–10% of women of reproductive age and 25–35% of those who experience infertility [1].

Endometriosis is a condition characterized by the detachment of endometrial tissue, including glands and stroma, from the uterine cavity. This detached tissue can be found in many locations such as the ovaries, peritoneum, utero-sacral ligaments, and uterine torus, as well as in areas of previous episiotomy or caesarean section scars. The presence of this detached tissue leads to the development of a persistent inflammatory response, thereby defining the condition of endometriosis [2]. This illness is characterized by infertility and persistent symptoms such as dysmenorrhea, dyspareunia, discomfort during ovulation, gastrointestinal problems, and dysuria [3].

The clinical symptoms of endometriosis exhibit a wide range of diversity. Consequently, the confirmation of an endometriosis diagnosis in women presenting with pelvic pain may need a considerable amount of time. The incidental detection of endometriosis might occur during a laparoscopic procedure or an imaging test. The kind and degree of symptoms are determined by the location of the endometriotic lesions and the affected organs [4]. Deep infiltrating endometriosis (DIE) refers to the presence of endometriotic lesions that surpass a depth of 5 mm beneath the peritoneal surface and invade the pelvic organs [5].

The diagnosis of DIE can provide greater challenges prior to surgical intervention due to its significant clinical variability. The evaluation of the condition should be conducted using non-invasive methods, taking into consideration the existing data. This can be achieved by integrating information from the patient’s medical history, clinical examination, imaging techniques, and treatment outcomes. Several diagnostic procedures are employed for the identification of endometriosis, including clinical examination, transvaginal ultrasonography (TVS), diagnostic laparoscopy, computer tomography, and magnetic resonance imaging (MRI) [6]. The diagnostic effectiveness of these approaches has been assessed in numerous research and from various perspectives.

TVS heavily relies on the expertise of operators, and it is essential that only medically trained personnel perform the procedure to ensure accurate diagnostic results [6,7]. The MRI technology offers the capability to effectively evaluate the complete peritoneal cavity. This method is non-invasive, albeit more costly, and serves as a means of detecting DIE [8]. The proficiency levels of radiologists and gynecologists have a significant role in the context of transvaginal ultrasonography and magnetic resonance imaging.

Standardizing the preoperative evaluation process and accurately diagnosing the patient prior to surgery can significantly improve their recovery and increase the likelihood of a successful surgical intervention, effectively removing all lesions and preventing the need for additional procedures.

The utilization of TVS as the principal modality for diagnosing individuals with suspected cases of deep pelvic endometriosis should be upheld [8]. In 2016, a group of researchers led by S. Guerreiro founded a group called the International Deep Endometriosis Analysis (IDEA) group. This group developed parameters to define the characteristics of DIE on TVS. This was developed in order to address the lack of standardized standards in the sonographic classification and diagnosis of DE [9]. Consequently, the researchers encourage the usage of TVS in a methodical manner, ensuring that endometriotic lesions are assessed using a standardized approach. It is crucial to employ consistent terminology to define the location and specific manifestations of DE, such as endometriomas, adenomyosis, and pelvic adhesions [9]. Previous research has made efforts to categorize endometriosis lesions based on ultrasonography characteristics; however, these attempts have not undergone external evaluation and have not been universally accepted [10].

MRI is often regarded as the imaging modality with the greatest overall precision in assessing the extent of endometriosis. It is typically employed as a secondary diagnostic approach following TVS to acquire a precise anatomical representation of the entire pelvic organs [5]. When conventional clinical examination and TVS are unable to detect abnormalities in individuals experiencing symptoms, MRI provides precise information for the staging of DIE, especially in cases involving parametrial lesions [11].

Lorusso et al. provided a comprehensive description of a standardized MRI methodology employed at their institution for the identification of DIE lesions. A consensus has not been established on the optimal approach on a global scale. The study team outlines the necessary criteria for obtaining highly precise outcomes in the identification of DIE lesions within their endometriosis methodology [5].

Bazot et al. presented a new set of criteria in the detection of DIE utilizing MRI, by requiring the application of sonographic gel to achieve opacification of the vaginal and rectal regions [12,13]. This promotes relaxation within the corresponding cavities, hence improving the visualization of the walls and potential endometriosis nodules, enabling the evaluation of the extent of the infiltration zone [12,14].

TVS is considered to be the simplest and most widely available diagnostic tool for endometriosis, as supported by previous research [6]. TVS is often the initial diagnostic imaging method used in patients with symptomatic DIE due to its cost-effectiveness and ease of access [9]. MRI, while possessing a high degree of reliability, is characterized by a greater cost compared to TVS, rendering it financially inaccessible for certain patients. The current healthcare system is unable to adequately cover the expenses associated with an MRI examination, as it is classified as an outpatient procedure. In addition, the utilization of vaginal and rectal gel during MRI procedures incurs higher costs compared to standard MRI investigations.

TVS can be conducted by a gynecologist during the initial consultation in any medical facility. It is important to highlight that the use of TVS is heavily reliant on the operator, and achieving accurate diagnostic results can only be accomplished by a proficient and knowledgeable medical staff, particularly in cases where an MRI examination is unattainable [6,7].

The primary aim of this study was to assess the diagnostic effectiveness of MRI and TVS in accurately determining the severity of endometriosis in patients prior to surgery.

## 2. Materials and Methods

### 2.1. Study Population

The present study is a retrospective longitudinal investigation of diagnostic accuracy. It was carried out over a span of four years, from January 2018 to August 2022, in private clinics and the “Prof. Dr. Panait Sîrbu” Obstetrics and Gynecology hospital in Bucharest, Romania. This hospital is a tertiary healthcare center that is affiliated with the “Carol Davila” University of Medicine, Bucharest, Romania. The patients included in our study met specific criteria for inclusion. These criteria consisted of clinical features that indicated the presence of endometriosis, such as symptoms like dysmenorrhea or dyspareunia, as well as findings from vaginal examinations that suggested the presence of a tender enlarged adnexal mass or palpable tender retrocervical or uterosacral ligament nodules. Additionally, patients were required to have undergone pelvic ultrasound examination or MRI that strongly indicated the presence of endometriosis.

All study participants have provided written consent for undergoing pelvic MRI and ultrasound imaging, as well as signed consent for the surgical treatment process and the collection of intraoperative histopathological samples. The study received approval from the medical ethics committee of the “Prof. Dr. Panait Sîrbu” Obstetrics and Gynecology hospital. Participants were deemed ineligible for inclusion in the study based on the following criteria: expressing a desire to be excluded from the study at any stage; lacking a signed informed consent for MRI, pelvic ultrasound imaging, and surgical procedure; not having a diagnosis of endometriosis or being pregnant; and virgin patients that couldn’t undergo a TVS examination.

### 2.2. Transvaginal Sonography

All patients underwent transvaginal sonography examinations conducted by a gynecologist with expertise in endometriosis at our clinic. The TVS examinations were performed using a vaginal 7.5 MHz probe (Voluson E8 Expert; GE Healthcare, Chicago, IL, USA). The evaluation of the patients was performed on nonmenstrual days of the menstrual cycle and under the condition of a partially filled bladder.

The TVS examination was conducted in accordance with the recommendations outlined by the IDEA group [9].

The assessment began by evaluating the central compartment, which includes the uterus and ovaries, identifying the existence of endometriomas or adenomyosis. Subsequently, the anterior and posterior compartments were examined for identification of endometriosis nodules [5,9]. Adnexal mobility and the “sliding sign” were examined in order to complete our TVS evaluation [9].

Through the identification of specific modifications in the density and shape of the afflicted organs, transvaginal ultrasound has the capability to detect endometrial implants and adhesions. Cysts, nodules, hypoechoic solid lesions, or other abnormal tissue growths, as well as peritoneal thickening, may be observed as a consequence of these modifications, suggesting the existence of DIE [5].

### 2.3. Magnetic Resonance Imaging

**Figure 1:**
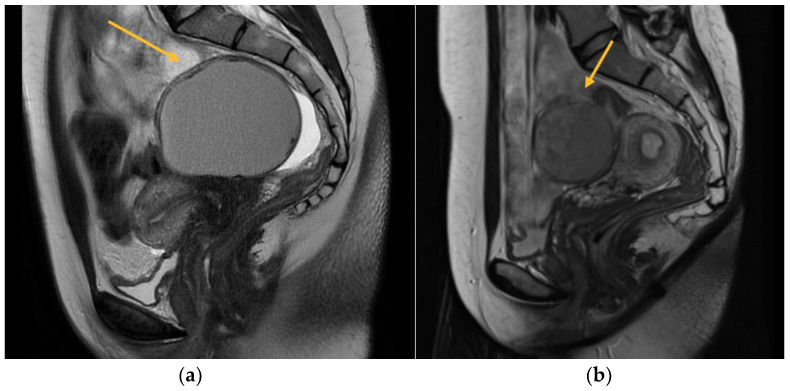
MRI aspect on T2 sagittal (**a**) Left endometrioma with left ovary adherent to the posterior uterine wall (**b**) Right endometrioma.

MRI is commonly utilized as an additional diagnostic method subsequent to TVS in order to obtain a precise anatomical representation of the pelvic organs. It has been found to possess the highest overall accuracy in detecting the extent of deep infiltrating endometriosis [5]. In the present study, a total of 256 patients were evaluated, out of which 177 underwent a 3.0 Tesla MRI scan. The exclusion criteria for MRI included personal factors such as claustrophobia, anxiety, and obesity. Additionally, patients who had already received a definitive diagnosis of endometriosis through TVS, those for whom an MRI examination was deemed unnecessary, and individuals with financial constraints limiting their ability to afford the high cost of the investigation were also excluded. To conduct a comprehensive assessment of the pelvic compartments and structures, a volume of 40 mcm^3^ of lubricant gel was introduced into both the vagina and rectum. This was done with the aim of augmenting the visualization and understanding of the pelvic anatomy. This approach enhances the perceptibility of the walls and potential endometriosis nodules, facilitating the quantification of the extent of infiltration (Figure 1).

The imaging techniques included axial, coronal, and sagittal T1- and T2-weighted images to accurately depict the anatomical structures and pathological features. Furthermore, the T1 axial and sagittal fat saturation techniques were performed, both with and without the use of contrast. The MRI assessments were read by a radiologist with specialized expertise in endometriosis, as the interpreter’s experience plays a crucial role in accurately diagnosing deep infiltrating endometriosis.

### 2.4. Laparoscopy and Histopathologic Evaluation

All 256 patients underwent laparoscopic interventions at our medical facilities, conducted by our surgical staff, under general anesthesia. The surgical interventions conducted aimed to remove all endometriosis lesions, which included the following procedures based on individual cases: excision of deep endometriosis nodules, ovarian cystectomy, salpingectomy, electrocoagulation of superficial endometriosis implants, recto-sigmoid resection with mechanical end-to-end anastomosis, and total hysterectomy (performed on six patients). The surgical procedure involving the gastrointestinal tract and urinary system necessitated the involvement of both a colorectal surgeon and a urologist.

The endometriosis lesions that were discovered and surgically removed during the operation were compared to those that were diagnosed using TVS and MRI. The histopathological assessment of the excised lesions conclusively verified the diagnosis of endometriosis in all examined samples. During the laparoscopic procedure, we classified each instance of endometriosis based on the new classification established by the American Society for Reproductive Medicine, as well as the #Enzian score and the Endometriosis Fertility index score (EFI) [15].

### 2.5. Statistical Analysis

All statistical computations were performed using the statistical analysis program SAS/STAT 14.2. Descriptive statistics for categorical variables were reported in the form of frequencies and percentages. The study employed the Chi-square test, Chi-square exact test, and Fisher’s Exact test to analyze the relationships between categorical variables. The evaluation of independence, accuracy, and concordance for categorical variables was conducted using Cohen’s kappa coefficient and McNemar’s test. The utilization of linear regression models was employed to investigate the associations between variables of a quantitative nature.

The findings are reported in relation to the average, variability, or proportions. The diagnosis of endometriosis was confirmed with certainty based on intraoperative findings and histological investigation. The intraoperative outcomes, as determined by the established diagnostic criteria, were compared to the findings obtained from preoperative imaging studies. The evaluation of diagnostic performance was conducted for each modality, taking into consideration the sensitivity, specificity, and accuracy. A *p*-value less than 0.05 is accepted as indicating statistical significance.

## 3. Results

The study examined various quantitative variables of the 256 endometriosis patients, including age, year of inclusion in the study, medical unit of treatment, symptoms experienced, clinical appearance, ultrasound imaging characteristics, MRI imaging characteristics, intraoperative appearance of lesions, surgical description, Enzian score, and EFI score. The diagnosis of DIE was confirmed in all 256 situations.

The patients’ median age was 34 years, with a mean age of 33.94 years. Based on the results of the Shapiro–Wilk test (*p* = 0.336), it can be inferred that the distribution of age categories was moderately balanced and adhered to a normal distribution.

The patients presented with symptoms including dysmenorrhea (100%, with varying Visual Analogue Scale values), dyspareunia (71.09%), and gastrointestinal issues (50.78%).

The study assessed the dysmenorrhea experienced by individuals with endometriosis by an anamnestic evaluation. This evaluation involved the use of the visual analogue scale (VAS), which allowed patients to subjectively rate the intensity of their pain. Participants provided self-reported pain intensity ratings on a numerical scale ranging from 0 to 10, where 0 indicated the absence of pain and 10 indicated the most severe pain experienced.

The majority of patients (37.11%) reported experiencing dysmenorrhea with a VAS score of 9. In contrast, a very small percentage (0.78%) of patients reported the lowest VAS score included in our study, which was 5. There were no instances when patients reported values below VAS = 5 or VAS = 6. The data is offered in Table 1.

**Figure 2:**
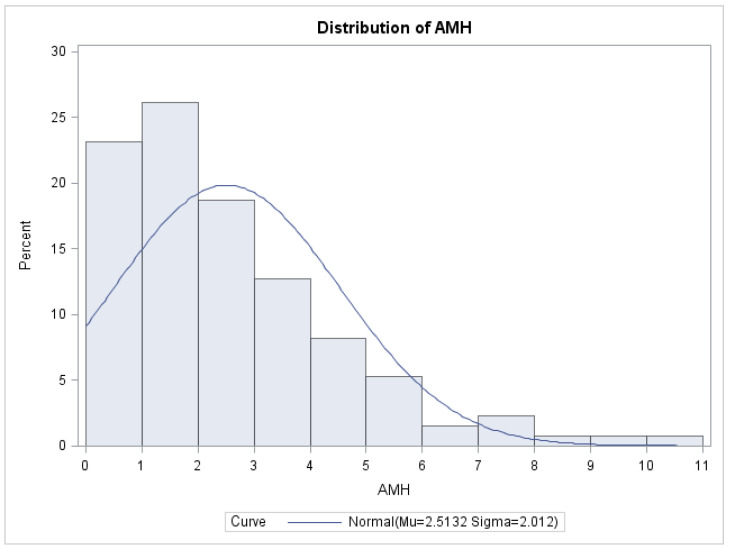
Distribution of AMH values.

A preoperative anti-Müllerian hormone (AMH) assessment was conducted on a subset of 134 individuals. The average AMH level was found to be 2.5 ng/mL, with a standard deviation of 2.01. The median AMH value was determined to be 2 ng/mL. The reference range for AMH levels in this study was established as 0.01–10.20 ng/mL. The AMH value measurement was not conducted for all 250 patients in the cohort due to consideration of the patient’s intention to conceive prior to the intervention. In instances where this desire was absent, there was no need to assess the ovarian reserve. Additionally, the measurement of AMH is not required in cases where the ovaries exhibit a normal ecostructure, characterized by the presence of many developing follicles and the absence of endometriomas. The data depicted in Figure 2 illustrates the distribution of anti-Müllerian hormone values among the 134 patients.

Before presenting to our clinic, 76 out of 256 patients had received medical treatment with combined oral contraceptives or progestins. Among the total of 256 patients, 38 individuals were diagnosed by TVS with adenomyosis.

The diagnostic precision of TVS and MRI in detecting DIE across several anatomical sites is succinctly presented in Table 2.

In this study, we conducted a comparative analysis of the diagnostic accuracy of transvaginal ultrasound and magnetic resonance imaging in various anatomical regions, with the aim of identifying the optimal diagnostic modality for evaluating DIE.

**Figure 3:**
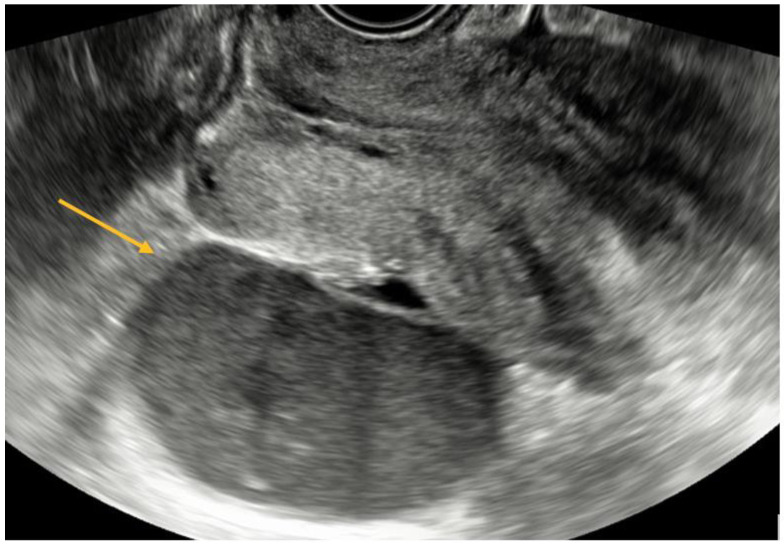
TVS aspect of endometrioma.

TVS is a reliable diagnostic tool for ovarian endometriomas (OMA), which can be readily identified due to their characteristic ground-glass appearance, which lacks any discernible vascular echo when assessed with a Doppler flow scanner, as depicted in Figure 3 [9,10].

The statistical analyses, namely the Chi-square χ^2^ test (df = 1) = 80.85, *p* < 0.001, and Fisher’s exact test *p* < 0.001, indicate a statistically significant connection. Therefore, within this cohort, the ultrasound diagnostic aligns with the intraoperative diagnosis for endometriomas. The specificity of the diagnostic test is 57.14%, indicating that it correctly identifies individuals without the condition. The sensitivity is 93.78%, indicating that it accurately detects individuals with the condition. The diagnostic accuracy, which is a significant measure, is 84.47%. The value of Cohen’s kappa coefficient was found to be 0.55, which indicates a moderate level of agreement. The statistical significance of this result was confirmed with a *p*-value < 0.001.

Among a total of 256 patients, it was observed that 193 individuals exhibited the presence of endometriosis cysts during intraoperative examination. Consequently, these patients had ovarian cystectomy. Among the observed cases, 39.37% exhibited left endometriotic ovarian cysts, 28.49% displayed right endometriotic ovarian cysts, and 32.12% presented with bilateral OMAs.

**Figure 4:**
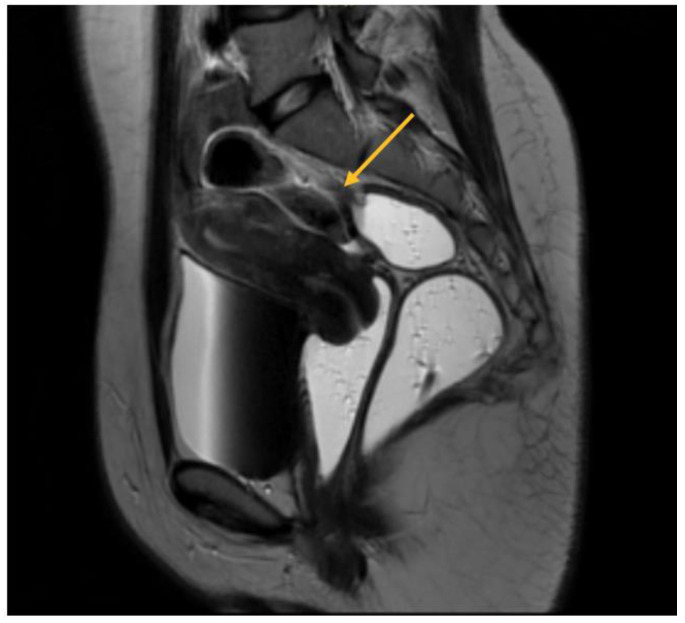
MRI aspect on T2 sagittal: parametrial endometriosis nodule.

In relation to parametrial lesions, the sensitivity of TVS and MRI was found to be 9% and 27.14%, respectively. The diagnostic accuracy of TVS and MRI exhibited similar levels of specificity. Specifically, TVS had a specificity of 97%, while MRI exhibited a specificity of 89.19%. Among a total of 256 patients, a significant majority of 189 individuals had parametrial lesions associated with intraoperative endometriosis. Consequently, the surgical intervention of laparoscopic excision was employed to address and remove these lesions. Among the observed cases, 31.21% exhibited left DIE parametrial lesions, while 20.63% displayed right DIE parametrial lesions. Additionally, 48.14% of the cases presented with bilateral lesions, as illustrated in Figure 4.

The precision of the two techniques was found to be 32% for TVS and 40.11% for MRI. The ultrasound diagnosis is not consistent with the intraoperative diagnosis for parametrial lesions of endometriosis, the association not being statistically significant according to the exact statistical tests Chi-square test and Fisher’s exact test (*p* = 0.172).

The MRI is consistent with the intraoperative diagnosis for parametrial lesions of endometriosis, the association being statistically significant according to the exact statistical tests Chi-square test and Fisher’s exact test (*p* = 0.049).

Specifically, for TVS, the Cohen’s kappa coefficient was calculated to be 0.03, with a *p*-value of 0.172. Similarly, for MRI, the Cohen’s kappa coefficient was determined to be 0.08, with a *p*-value of 0.049.

As will be elaborated upon in the forthcoming discussion section, the identification of parametrial lesions may prove more challenging in the absence of collaboration with a radiologist possessing specialized expertise in recognizing DIE lesions. The parametrial nodule manifests as a hypointense thickening or evidence of a mass with irregular contours situated in the posterior uterine region, as depicted in Figure 4 [16].

Regarding USL, the sensitivity of TVS was significantly lower compared to MRI, with rates of 14.63% and 65.88%, respectively. The specificity of TVS was found to be higher compared to MRI, with values of 94.74% for TVS and 66.30% for MRI. Among a total of 256 patients, it was observed that 123 individuals exhibited endometriosis lesions of the uterosacral ligaments during intraoperative examination. Consequently, laparoscopic excision was conducted to remove these lesions.

Among the observed cases, it was shown that 31.71% exhibited lesions in the left USL, while 30.89% displayed lesions in the right USL. Additionally, 37.40% of the cases revealed bilateral lesions in the USL. The accuracy of the two procedures was as follows: 55% for TVS, which was found to be statistically significant (Cohen’s κ = 0.09, *p* = 0.019), and 66.1% for MRI, which was found to be statistically significant (Cohen’s κ = 0.32, *p* < 0.001).

**Figure 5:**
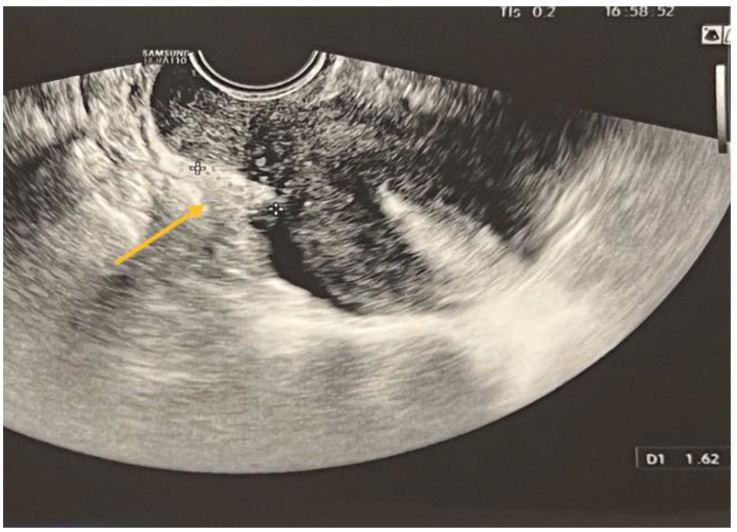
TVS aspect of uterine torus and USL endometriosis lesions.

USL endometriosis involvement is usually diagnosed by TVS when assessing the posterior pelvic compartment. USL lesions appear as depicted in Figure 5, as a linear hypoechoic thickening exhibiting either regular or irregular edges [9]. Particularly, we have noticed the nodule appears to have a high-echoic appearance compared to what is commonly described in the relevant literature. However, it is important to highlight that the contour and demarcation of the surrounding structures are clearly discernible.

USL lesions are usually linked to concurrent DIE lesions, the arrow in Figure 5 indicating the presence of a USL nodule connected to a uterine torus DIE lesion. The section indicated by asterisks represents the measurement of the length of the lesions.

In the case of rectal endometriosis, the specificity of TVS was shown to be lower compared to MRI, with values of 76.87% and 94.51%, respectively. The sensitivity of TVS and MRI exhibited a similar level of comparability, with values of 69.72% and 66.28%, respectively. The diagnostic accuracies of the two procedures were found to be similar and statistically significant. For TVS, the accuracy was 73.82% (Cohen’s κ = 0.46, *p* < 0.001), while for MRI, the accuracy was 80.79% (Cohen’s κ = 0.61, *p* < 0.001). Among a total of 256 patients, 109 individuals exhibited rectal endometriotic nodules, leading to the implementation of surgical procedures. Specifically, rectal shaving was performed in 5.5% of cases, discoidal resection in 1.84% of cases, and end-to-end anastomosis segmental resection in 92.66% of cases.

**Figure 6:**
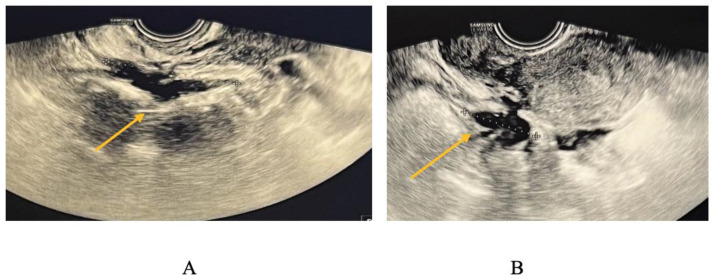
(**A**) TVS aspect of rectal endometriosis lesion, (**B**) TVS aspect of rectal endometriosis lesion adherent to uterine torus endometriosis lesion.

TVS is employed to diagnose bowel DIE by recognizing the thickening of the intestinal wall in a solid or plaque-like approach, as well as the absence of visceral fat separating the recto-sigmoid and the uterine wall [17,18]. The findings are depicted in Figure 6, where the yellow arrow highlights the existence of a rectal nodule exhibiting a negative “sliding sign” and the asterisks indicate the length of the lesion. This sign denotes the absence of rectal mobility in relation to the uterus and the posterior vaginal fornix, suggesting the likelihood of adhesion and endometriosis lesions [17,18]. The adherence of the rectal DIE nodule to the uterine torus is illustrated in Figure 6B.

**Figure 7:**
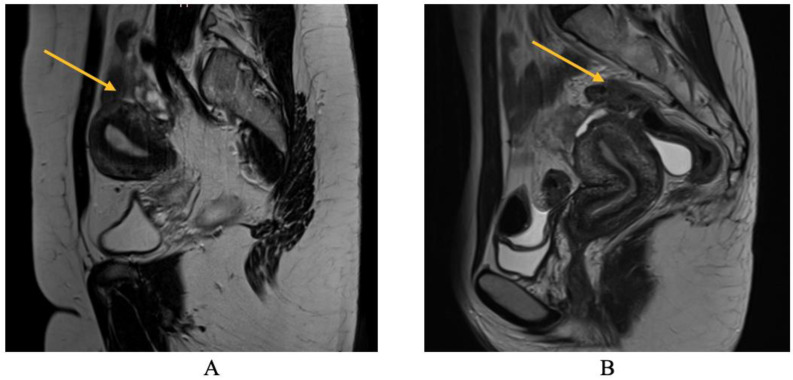
MRI aspect on T2 sagittal (**A**,**B**): recto-sigmoidian endometriosis nodule adherent to the uterine fundus via adenomyosis lesion.

Regarding MRI diagnosis of bowel DIE nodules, Figure 7 illustrates the presence of nodular thickening, which leads to anatomical deformation of the intestinal loop. The rectal nodule is adherent to the posterior uterine wall through an adenomyosis lesion, as indicated by the yellow arrow. Rectal DIE nodules typically exhibit adherence to other DIE lesions, including uterine torus, USL, parametrial, or adenomyosis [13,14].

The study yielded a sensitivity rate of 58.33% and a specificity rate of 96.73%, resulting in an overall accuracy of 93.22%. These findings were found to be statistically significant, as indicated by a Cohen’s κ value of 0.60 (*p* < 0.001).

## 4. Discussion

Endometriosis specialists possessing substantial expertise in the identification, therapeutic intervention, and overall handling of the disease are more inclined to identify DIE lesions prior to surgical intervention. When comparing a gynecologist who lacks regular evaluation of DIE lesions and the ability to integrate imaging findings for accurate diagnosis, a gynecologist with training and expertise in TVS is more proficient in identifying endometriosis lesions. The primary determinant in evaluating the severity of endometriosis is the proficiency of the examiner.

The non-invasive diagnosis of DIE can be achieved by integrating data from the patient’s medical records, clinical examination, imaging procedures, and treatment outcomes, while also considering the existing literature in the field.

In our study cohort of patients presenting with symptoms of endometriosis, it was observed that MRI exhibited superior diagnostic accuracies compared to TVS, notably in the evaluation of the rectovaginal and parametrial regions.

The utilization of transvaginal ultrasound is considered the optimal diagnostic modality for the identification of ovarian endometriosis cysts [5,9]. This approach is preferred due to its accessibility, reliability, and ability to provide a distinct ultrasound appearance that is sufficient for the accurate diagnosis of endometriomas. In cases when ultrasound results show the presence of ovarian endometriosis without evidence of extensively infiltrative endometriosis lesions, it is our recommendation that an MRI assessment may not be necessary.

The specialized research discovered that both TVS and MRI demonstrated similar levels of sensitivity and specificity in diagnosing endometriomas. The sensitivity values were from 70.86% to 96% for TVS and from 63.5% to 92.6% for MRI. Additionally, the specificity values ranged from 71% to 96% for TVS and from 71% to 93.9% for MRI [19,20,21].

TVS is a reliable diagnostic tool for endometriomas, obviating the necessity for MRI confirmation, as a result of our study as well.

Based on expert study, it has been shown that the accuracy of MRI diagnosis for parametrial endometriosis lesions is 96.4%, with a specificity of 98.6% and a sensitivity of 83.3% [19]. Furthermore, our research demonstrates a statistically significant association between the diagnosis of parametrial endometriosis lesions through MRI and their emergence during surgical procedures.

Based on our research findings, there is a lack of agreement between the intraoperative diagnosis of parametrial lesions associated with endometriosis and the ultrasound diagnosis of these lesions.

Barra et al. propose distinct ultrasonographic landmarks and indicators to identify the presence, particularly the coexistence, of parametrial endometriosis lesions alongside other deep endometriosis lesions. These findings are documented in the relevant scientific literature [19]. According to sonographic findings, certain characteristics such as the absence of a posterior sliding sign, the presence of multiple ovarian endometriosis cysts with the affected ovary adhering to the posterior surface of the uterus, the existence of retro-cervical endometriosis nodules or nodules at the level of the uterine torus, the occurrence of hydronephrosis, the infiltration of the rectovaginal septum, or the presence of recto-sigmoid nodules are all indicative of the potential for parametrial invasion [16,19].

In order to further our research and improve the sonographic diagnosis of parametrial endometriosis, we aim to see any of these indicators through sonographic examination in patients who have an MRI diagnosis or clinical suspicion of parametrial endometriosis.

According to the existing literature, it is widely acknowledged that MRI has a higher sensitivity in identifying endometriotic lesions located in the USL compared to TVS evaluated to the evaluation of TVS. Specifically, MRI demonstrates a sensitivity range of 63.5% to 95.6%, whereas TVS shows a sensitivity range of 55.6% to 78.3%. The specificity levels of TVS, ranging from 66.7% to 98%, and MRI, ranging from 60% to 93.9%, exhibit similar values [17,20,21,22,23,24,25,26].

The findings presented in this study exhibit a high degree of alignment with the existing body of research. The findings of our study indicate that in cases when there is suspicion of deep infiltrative endometriosis, an MRI examination is necessary due to its significantly greater sensitivity compared to a TVS examination.

Based on data extracted from academic literature, it has been reported that MRI exhibits a sensitivity ranging from 76.9% to 94% and a specificity ranging from 50% to 96.6% in the context of diagnosing recto-sigmoid intestinal endometriosis lesions [17,20,22,23,25]. The findings of our study demonstrate a high level of concordance between the MRI diagnosis of rectal nodules and the intraoperative diagnosis.

The findings of our research demonstrate a statistically significant association between the diagnosis of sigmoid nodules through MRI and intraoperative diagnosis, as well as between intraoperative diagnosis and ultrasonography. There is a potential for misidentification of sigmoid nodules as rectal nodules with the application of ultrasonography for the detection of rectal endometriosis. Specialized studies have proposed the utilization of transrectal ultrasonography for a more comprehensive evaluation of the intestinal involvement in endometriosis. Nevertheless, MRI has demonstrated a high diagnostic accuracy in detecting these lesions [25]. Magnetic resonance imaging is the preferred diagnostic modality for sigmoid endometriosis, as it has been observed that these nodules may be prone to misdiagnosis or underdiagnosis.

The diagnostic accuracies of various approaches vary significantly among studies, primarily due to differences in methodologies and expertise employed. The diagnostic accuracy varies across different anatomical locations.

The initial imaging evaluation that should be conducted on individuals who might suffer from endometriosis is TVS. The combination of MRI and TVS is recommended for individuals who present with unexplained pelvic pain [18,21].

MRI exhibits superior accuracy compared to TVS in diagnosing DIE. Consequently, the achievement of effective diagnostic results necessitates the involvement of a proficient and well-trained medical team [7,22].

To optimize the comprehensive execution of surgical therapy, the selection of techniques relies on the expertise of the gynecologist and radiologist during the preoperative assessment of the lesions.

Scientists are consistently making advancements in the development of innovative and minimally invasive methods for the diagnosis of endometriosis. One noteworthy aspect to address is the integration of artificial intelligence (AI) in performing TVS and/or MRI examinations [26]. AI has been evaluated in numerous endometriosis-related studies for predicting outcomes and building diagnostic models using plasma biomarkers, serum miRNA markers, and classifying endometriosis through employing the technique of peptide profiling [26,27]. AI has the potential to effectively contribute to the development of a diagnostic algorithm that integrates physical symptoms, imaging modalities such as TVS, MRI, and three-dimensional reconstructions, as well as the history of infertility. This integration has the capacity to minimize diagnostic delays and enhance the efficacy of treatments, hence improving overall treatment outcomes [27].

Strain elastography is an additional diagnostic modality employed for the evaluation of endometriosis. Xholli et al. discuss the utilization of elastography, a technology with the ability to quantitatively assess tissue structure and elasticity, in the identification or the monitoring of the progression of established DIE nodules [28]. Brunelli et al. emphasize the significant role of elastography in the accurate diagnosis of deep pelvic endometriosis lesions, exhibiting high sensitivity and specificity [29].

Three-dimensional (3D) reconstructions, comparable to those employed in the field of fetal medicine, are used to diagnose DIE lesions via TVS or MRI investigations [30,31]. The integration of 3D MRI reconstructions has yielded enhancements in the reliability of diagnosing DIE [31]. This advancement facilitates a meticulous preoperative mapping of these lesions. However, the incorporation of 3D reconstructions into TVS does not yield any significant enhancements in the diagnostic accuracy of TVS for detecting endometriosis nodules [31].

Leonardi et al. propose the usage of saline-infusion sonoPODography (SPG) for diagnosing superficial endometriosis, given that laparoscopy currently represents the best available approach for this purpose [32]. SPG has demonstrated its potential in the estimation of the ASRM endometriosis classification and the Endometriosis Fertility Index [32].

## 5. Conclusions

The present study aimed to evaluate the diagnostic effectiveness of two non-invasive methods, transvaginal ultrasound and magnetic resonance imaging for the detection of deep infiltrating endometriosis. While laparoscopy and histopathologic evaluation of samples have been widely regarded as the preferred method for identifying DIE, it is important to acknowledge that both procedures are invasive in nature and need preoperative preparation. Both TVS and MRI are widely used and easily accessible non-invasive methodologies. The early identification of DIE has the potential to accurately predict the prognosis, quality of life, and reproductive function.

Therefore, the results of the present study indicate that TVS exhibits a diagnostic accuracy for the identification of DIE that is, to a certain extent, comparable to that of MRI. TVS is the preferred diagnostic modality for endometriomas, and it exhibits a similar level of accuracy when assessing recto-sigmoid DIE nodules.

In instances where TVS proves insufficient or impracticable, the gynecologist needs to schedule for MRI to be conducted. Both modalities are contingent upon experience, and it is the interpreter who determines their appropriate utilization.

Comprehensive preoperative assessments have the potential to decrease the incidence of incomplete surgeries resulting from inadequate understanding of the illness’s vast breadth and complexity. Additionally, such assessments can facilitate appropriate referrals to specialized institutes.

## Figures and Tables

**Table 1:** Distribution of dysmenorrhea according to VAS value–descriptive statistics parameters.

Dysmenorrhea (VAS)	Number of Patients	Percentage
5	2	0.78%
7	19	7.42%
8	54	21.09%
9	95	37.11%
10	86	33.59%

**Table 2:** The diagnostic accuracy of TVS and MRI for DIE locations.

Endometriosis Lesions	Laparoscopic Findings			Sensitivity	Specificity	Diagnostic Accuracy
	+	−	Total			
OMA				93.78%	57.14%	84.47%
TVS +	181	27	208			
−	12	36	48			
Total	193	63	256			
Parametrial DIE				9%	97%	32%
TVS +	17	2	19			
−	172	65	237			
Total	189	67	256			
MRI +	38	4	42	27.14%	89.19%	40.11%
−	102	33	135			
Total	140	37	177			
USL DIE				14.63%	94.74%	55%
TVS +	18	7	25			
−	105	126	231			
Total	123	133	256			
MRI +	56	31	87	65.88%	66.30%	66.1%
−	29	61	90			
Total	85	92	177			
Rectal DIE				69.72%	76.87%	73.82%
TVS +	76	34	110			
−	33	133	146			
Total	109	147	256			
MRI +	57	5	62	66.28%	94.51%	80.79%
−	29	86	115			
Total	86	91	177			
Sigmoidian DIE				58.33%	96.73%	93.22%
MRI +	14	5	19			
−	10	148	158			
Total	24	155	177			

OMA—Endometrioma, TVS—Transvaginal ultrasound, MRI—Magnetic resonance imaging, USL—Uterosacral ligaments, DIE- deep infiltrating endometriosis.

## Data Availability

The data used to support the findings of this study are available upon request to the corresponding author.
